# Individual Placement and Support and Participatory Workplace Intervention on the Work Participation of People with Disabilities: A Randomised Controlled Trial

**DOI:** 10.1007/s10926-024-10219-6

**Published:** 2024-07-02

**Authors:** E. Oude Geerdink, M. A. Huysmans, H. van Kempen, J. van Weeghel, E. Motazedi, J. R. Anema

**Affiliations:** 1 Department of Public and Occupational Health, Amsterdam Public Health Research Institute, Amsterdam UMC - Location VU Medical Center, Vrije Universiteit Amsterdam, Van der Boechorstraat 7, Postbus 7057, 1081 BT Amsterdam, The Netherlands; 2 Research and Statistics, City of Amsterdam, Amsterdam, The Netherlands; 3 Tranzo, Tilburg School of Social and Behavioral Sciences, Tilburg University, Tilburg, The Netherlands

**Keywords:** Vocational rehabilitation, Labour market participation, Occupational health, Supported employment, RCT, Social welfare

## Abstract

**Purpose:**

This study assessed the effectiveness of Individual Placement and Support (IPS), Participatory Workplace Intervention (PWI), and IPS + PWI on work participation and health of people with work disabilities.

**Methods:**

A randomised controlled 2 × 2 factorial trial with 120 clients and an 18-month follow-up was performed. Differences between IPS and no-IPS and between PWI and no-PWI were assessed using log-rank tests and Cox proportional hazards models.

**Results:**

In the IPS group, restricted mean survival time (RMST) for sustainable paid employment was 352 days, compared to 394 in the no-IPS group (HR = 1.47, 95% CI = 0.81–2.63). In the PWI group the RMST was 378 days, compared to 367 in the no-PWI group (HR = 0.89, 95% CI = 0.48–1.64). For the secondary outcome ‘starting any paid employment, a trial placement, or education’ RMST was significantly lower for the IPS group (222 days) than for the no-IPS group (335 days; HR = 1.85, 95% CI = 1.01–3.42). Mental health was significantly lower (worse) in the PWI group (difference -4.07, 95% CI = -7.93 to -0.22) than in the no-PWI group. For all other secondary outcomes, no statistically significant differences were found.

**Conclusion:**

No statistically significant differences were observed in the duration until starting sustainable employment between IPS and no-IPS, and between PWI and no-PWI. The duration until starting any paid employment, a trial placement, or education was shorter in the IPS group than in the no-IPS group, but further research should explore whether this also increases sustainable employment in the longer term.

**Supplementary Information:**

The online version contains supplementary material available at 10.1007/s10926-024-10219-6.

## Introduction

For most people work represents an important aspect of their lives: having a paid job provides financial independence, serves as a source of identity, and can increase health and wellbeing [1–3]. On the other hand, unemployment can negatively affect health and wellbeing [4, 5]. Finding and keeping a job is more difficult for some people than for others: labour market participation among people with work disabilities is low compared to among people without work disabilities [6, 7]. In the context of this paper, the term “people with work disabilities” refers to people facing physical, psychological and/or social limitations that hinder their ability to find and maintain employment [8–11]. People with work disabilities often depend on agencies such as municipalities or social security offices to receive support in finding and keeping a job. It is important that the services of these agencies are evidence based, to increase their effectiveness.

The Individual Placement and Support (IPS) model of supported employment is an intervention that has been proven to increase labour market participation for people with work disabilities caused by mental health problems, specifically for people with severe mental illness (SMI) [12–15]. IPS mainly distinguishes itself from other types of employment support through the integration of employment services with health care services [12]. Another key element of IPS is the ‘zero exclusion’ principle, which means that every client can start an IPS trajectory if they wish to, regardless of the severity of their symptoms [12]. IPS uses the ‘first place, then train’ principle, prioritising participation in regular paid work and providing training at the workplace if necessary [12]. The effectiveness of IPS for people with SMI has since long been established, but more recently it has also been applied for other populations [16–18]. IPS has been found promising to improve work participation for veterans with spinal cord injury [18, 19], and a systematic review found strong evidence for the effectiveness of IPS for veterans with posttraumatic stress disorder [16]. An RCT in Norway that investigated whether IPS can be effective for young adults at risk of early work disability also found that IPS was more effective than the usual service for finding paid employment [17]. Finally, a recent meta-analysis showed that IPS was also effective for people with common mental disorders (CMDs) [20]. Therefore, it is worth investigating whether IPS can also improve the effectiveness of welfare-to-work services for people with work disabilities which, as mentioned previously, can consist of a combination of physical, psychological and/or social limitations.

A second method that might be effective in increasing work participation of people with work disabilities is a Participatory Workplace Intervention (PWI) [21]. PWI is a conversation method that takes place in the workplace and uses a stepwise approach, in which the employee and their supervisor reach consensus on the most important obstacles for work functioning and on solutions that can be implemented to increase the sustainability of employment. PWI interventions have also been applied to multiple populations in previous research, and were especially effective for people with musculoskeletal disorders [22–24]. Studies that included participants with mental health disorders usually found limited or no effects, but in most of these studies this was explained by a low adherence to protocol [25–27]. Nevertheless, PWI might be a suitable intervention for people with work disabilities, because it can be used to systematically address the problems that these people may experience in multiple areas of life [8]. Since these problems can play a role in continuing employment, using PWI may especially increase the chances of sustainable employment.

By designs and working mechanisms, IPS and PWI could be applied during the same welfare-to-work trajectory. IPS focuses on finding competitive work and provides coaching at the workplace. As part of this coaching, PWI can be used to improve the sustainability of the placement. Both as standalone interventions and when provided to the same client, IPS and PWI have the potential to increase sustainable employment for unemployed people with work disabilities. Therefore, we performed a randomised controlled trial (RCT) to examine the effectiveness of IPS and PWI for reducing the duration until sustainable work participation among people with work disabilities. Furthermore, we examined the effects of IPS and PWI on additional work outcomes, societal participation, and physical and mental health.

## Methods

### Study Design and Context

An RCT with a 2 × 2 factorial design was performed to evaluate the effectiveness of IPS and PWI in reducing the time until starting sustainable work for people with work disabilities. Sustainable work was defined as working for at least 28 days consecutively, for at least 12 hours per week on average. The follow-up was 18 months. The trial was conducted within the department of Work and Participation in a large municipality in the Netherlands. The context of the Dutch social welfare system is explained in Box 1. The Medical Ethics Committee of the VU University Medical Centre approved the study design and declared that it was not subject to the WMO (Medical Research Involving Human Subjects Act, no. 2018.462)*.* All participants signed the approved informed consent form before inclusion in the trial. The project was registered in the Dutch Trial Register (NL9771) and can be found on the International Clinical Trial Registry Platform (ICTRP).

**Box 1:**
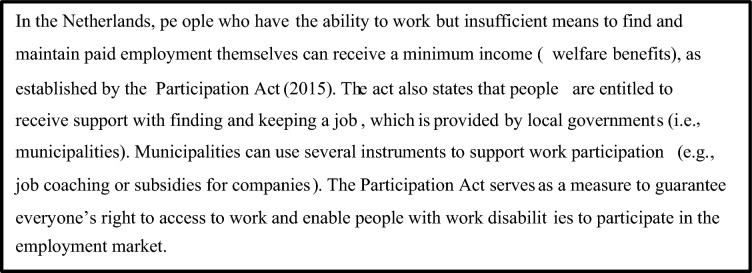
Description of the Dutch social welfare system

### Eligibility Criteria

Clients were eligible to participate in this study if they (i) were recently referred to the Work Disabilities team, (ii) were willing to obtain competitive work, (iii) had a (suspected) work disability, and (iv) were 16 years or older. A work disability was defined in line with the definition that was used within the municipality the study took place in, which was: ‘not being able to provide your own income due to long-term (i.e. not expected to be solved within a year) physical, psychological, and/or social problems.’ Exclusion criteria were (i) being unable to give informed consent due to cognitive or language barriers; (ii) already having competitive employment for 12 h or more per week.

### Recruitment, Randomisation, and Blinding

Recruitment took place between November 2019 and October 2021 (due to COVID-19 restrictions, recruitment was paused for approximately six weeks in March 2020 and was then continued remotely). All clients who were referred to the Work Disabilities team during this period were approached by a researcher by telephone. When clients were eligible and wanted to participate, an appointment was made in which the informed consent form was signed and the baseline questionnaire was completed. After that, participants were randomly assigned to IPS or no-IPS (1:1 ratio) and at the same time to PWI or no-PWI (1:1 ratio). The randomisation was stratified by age group (adolescents 16–26, and adults older than 26) for practical reasons; adolescents and adults were coached by different teams with dedicated job coaches. Randomisation was also stratified by work readiness groups (those who were considered ‘ready to work’ and those who were considered ‘not yet ready to work’). For each stratum a randomisation sequence was generated by a statistician who had no further involvement in the study, using block randomisation with varying block lengths. Blinding of clients and job coaches was not possible due to the nature of the interventions.

### Interventions

IPS, PWI, and Service as Usual (SAU) were provided by job coaches who worked for one of the Work Disabilities teams. All job coaches who participated had at least 2 years of experience with coaching people with work disabilities to find and keep a job. For practical reasons job coaches were not randomised, but allocated to one of the trial arms based on their preference and skills. Usually when clients of job coaches in the municipal setting start a new job, their job coach supports the client and employer in the application process and discusses potential coaching needs or workplace accommodations that may be necessary. The job coach is also responsible for explaining the rules and regulations to the employer and for providing them with information on subsidies. The job coach is therefore usually in close contact with the employer and/or a direct supervisor. For this trial, job coaches did not have to change this part of their usual way of working for clients who received IPS or SAU. Also employers and supervisors were not expected to do something different for the IPS intervention than adhere to general good employer practices and facilitate reasonable accommodations in the workplace when necessary. For PWI direct supervisors were expected to be involved in the conversations at the workplace, which were more extensive than with SAU. Job coaches informed employers of PWI-clients about the intervention and their roles as soon as possible after they started working.

#### Individual Placement and Support (IPS)

Eight principles describe the aims and method of IPS [28]: first, there is a focus on competitive employment. Second, the zero exclusion principle means that everyone who wants to work receives coaching, regardless of symptoms or work experience. Third, within the job search attention is on clients’ preferences. Fourth, the search for a job starts immediately, without prevocational training. Training can take place at the workplace if necessary. This is according to the ‘first place, then train’ principle. The fifth principle is targeted job development, which means that IPS specialists actively build on their own network of employers. The sixth principle describes the integration of employment services with mental health treatment teams. The seventh principle of personalised benefits counselling ensures that IPS coaches provide clients with information on how working impacts their financial situation. The eighth principle is individualised long-term support, which provides clients with individual coaching for as long as they want and need.

Job coaches who participated in the IPS group were trained to become certified IPS coaches. This training was an adapted version of the regular IPS training that is commonly provided in the Netherlands. The training was provided by a certified IPS trainer and consisted of two days of theory, 3 supervision group meetings, a one-on-one meeting between the job coach and the IPS trainer, and a closing session. As in the regular IPS trainings, job coaches had to prepare assignments for each meeting and hand in a report before the closing session. The supervision group meetings and one-on-one meetings consisted mainly of discussing cases and dilemmas that job coaches encountered in practice. Because job coaches already had sufficient experience with job coaching and knowledge on rules and regulations, certain aspects of the training were limited and, therefore, two days of theory was sufficient, compared to the usual 3 days of theory. Because IPS took place in a municipality rather than in a healthcare setting, the training was further adapted to better suit the needs of job coaches. More attention was paid to collaboration with (mental) health care services, which was complicated given the unusual setting. Job coaches were instructed to contact important care providers and family members or friends of their clients, if the client gave permission. The main goal of these contacts was to agree on the goals of coaching and thereby prevent clients from receiving conflicting advice.

#### Participatory Workplace Intervention (PWI)

PWI is a conversation method that is guided by a process leader and consists of a structured stepwise process. The main goal is for clients and their supervisors to identify obstacles for sustainable employment and implement solutions to solve these obstacles. PWI consists of three steps: in the first step, the process leader performs a task analysis and identifies obstacles for work continuation with the client and with the supervisor separately. The task analysis consists of filling in a matrix in which all work tasks are described. For each task it is assessed whether obstacles are experienced and if so, how often they occur and how serious they are [22]. In the second step, the process leader initiates a conversation between the client and supervisor together, in which they reach consensus on which obstacles are most important. Next, they brainstorm about solutions and then the most suitable solutions are put into an action plan. This plan describes who will do what, when, and how. In the third step, an evaluation is done to determine whether the steps of the action plan were put into practice and whether that solved the obstacles. The total duration of the intervention is dependent on the timing of the steps in the action plan. Although PWI typically starts after a job was found, process leaders in this study could use a ‘preparatory PWI’ while searching for a job. This is an optional part of PWI in which similar steps can be completed by the client and process leader, to identify and solve obstacles for starting paid employment.

Before the start of the trial participating job coaches received a four-hour training to become PWI process leaders. The intervention and training materials for PWI were specifically designed for this study, by tailoring the materials of previously described participatory workplace interventions to this setting [22, 29]. The training was provided by JRA and MAH, who both had experience with providing similar participatory workplace intervention trainings. An additional training took place a couple of weeks after the recruitment of participants started, because job coaches indicated a need for a ‘refresher course.’ The trainings provided theory, that was practised by putting the intervention in action through role play. As in the IPS group, monthly intervision meetings took place between the start of inclusion of participants and the moment all participants were included in the study for at least one year.

#### IPS + PWI

Given the factorial design, several job coaches were trained in both IPS and PWI and provided their clients with both interventions. The goal of IPS + PWI was to obtain sustainable employment by providing clients with both methods. The principles of IPS were followed, and when clients started working PWI was carried out as part of the coaching at the workplace. The preparatory PWI could be used as well, as part of the intake and assessment phase of IPS. Job coaches who participated in the IPS + PWI group followed the IPS training together with job coaches in the IPS group, and received a separate PWI training in which extra attention was paid to when to initiate PWI during the phase of coaching at the workplace. The monthly intervision meetings for job coaches who provided IPS and PWI were separate from those who provided only IPS or only PWI.

#### Service as Usual (SAU)

SAU entailed that clients were first evaluated based on their readiness to work. Clients who were considered ‘not yet ready’ received coaching based on the ‘first train, then place’ principle. This entails that they first received prevocational training, which could consist of education or traineeships. When they were then considered ‘ready to work’ they were assigned to a job coach. Clients who were directly considered ‘ready to work’ were assigned to a job coach immediately. The task of the job coach was to guide the client in what was needed to find a job. They sometimes collaborated with job hunters, whose task it was to find a suitable job using their elaborate network of employers. The duration and intensity of the SAU was dependent on the needs of the client.

### Outcomes and Data Collection

#### Primary Outcome and Secondary Work-Related Outcomes

The primary outcome ‘duration until starting sustainable paid employment’ was operationalized as the number of days between inclusion in the study and the start of participation in paid employment for at least 28 days, and at least 12 hours per week on average. We used non-public microdata from Statistics Netherlands (CBS). Sheltered employment was excluded from the primary outcome, but paid employment in so-called ‘social job companies’ was not excluded. In social job companies, people with work disabilities receive a regular salary and mostly work in regular work environments (i.e. a cleaning job in a regular company). However, there is generally more coaching at the workplace, jobs can often be more tailored to the individual, and often direct colleagues also have a work disability.

Additional employment-related outcomes were collected from CBS, complemented with information from the registries of the municipality about education and trial placements. A trial placement means that a client starts working for an employer who has an intention to hire the client, but the client receives welfare benefits instead of wages during the trial period [30]. Trial placements are often used to persuade employers to hire clients with a work disability by minimising financial risks for the employer. The duration of a trial placement is typically two months, after which paid employment starts [30].

Secondary work-related outcomes were:

- Any paid employment (≥ 1 h) during 18-month follow-up: a dichotomous variable was created indicating whether any payment from employment had been received since inclusion in the programme (based on CBS data).
- Duration in days from inclusion until starting any paid employment (based on CBS data).
- Duration in days from inclusion until starting any paid employment, a trial placement, or regular education (based on CBS and registration data).
- Duration in days from inclusion to starting sustainable paid employment (≥ 12 h per week on average) for at least three months consecutively (based on CBS data).
- Duration in days from inclusion to starting sustainable paid employment (≥ 12 h per week on average) for at least six months consecutively (based on CBS data).
- Total number of hours worked in paid employment between inclusion in the study until 18-month follow-up (based on CBS data).

#### Societal Participation and Health-Related Outcomes

Additional secondary outcomes were collected through questionnaires filled in by clients at baseline, six months, and twelve months after enrolment. Participants either filled in the questionnaire themselves, with a friend or family member, or with one of the researchers (face-to-face or during a telephone call), depending on their preference.

Additional non work-related secondary outcomes were:

- Level of societal participation, determined by job coaches using the ‘participation ladder’ that can be scored on a range from 1 to 6 [31].
- Mental and physical health, measured with the Dutch 12-Item Short Form Health Survey (SF12). Two summary scores (physical (PCS) and mental (MCS) with a standard mean of 50 were calculated [32, 33].
- Perceived ability to participate in social roles and activities, and satisfaction with this ability, measured with the PROMIS Short Forms 8a, version 2, *‘ability to participate in social roles and activities’* and *‘satisfaction with social roles and activities’* [34]. T-scores with a standard mean of 50 were calculated.
- Perceived work ability, i.e. the extent to which clients feel they are capable to work, measured in the client questionnaire at baseline, six-month, and twelve-month follow-ups, with one question that could be answered on a scale of 1 to 10: *“If you would give your (psychological and physical) ability to work 10 points in the best period of your life, how many points would you give it at this moment?” *[35, 36]*.*

### Sample Size

Based on previous studies, we considered a hazard ratio (HR) of two between an intervention group and its control group as the minimal clinically relevant effect on the primary outcome [24, 37]. Assuming that 50% of clients start sustainable paid employment after one year and there is a dropout rate of 10%, a total number of 60 clients was needed in each of the IPS/no-IPS and PWI/no-PWI groups to achieve 80% power at a two-tailed significance level of $\alpha =0.05$ using the log-rank test. In order to take into account the clustering effect of the job coaches treating several clients, power calculations were based on intensive simulations with varying RTW percentages per job coach with an intraclass correlation coefficient (ICC) of 0.05.

### Statistical Methods

Descriptive statistics were used to describe the baseline characteristics of the different study groups. Baseline differences between groups were analysed using Fisher’s exact or Fisher–Freeman–Halton exact tests for categorical variables, and independent samples t tests for continuous variables. For the primary outcome, the Cox proportional hazards model was used to assess treatment effects of IPS versus no-IPS and PWI versus no-PWI by estimating hazard ratios (HRs) and their corresponding 95% confidence intervals (CIs). To take the possible clustering effect of the job coaches into account, a robust ‘sandwich’-type estimator of the coefficients covariance matrix was used to determine the standard errors and CIs of the estimated HRs [38]. The significance of each treatment's effect was also tested using the log-rank test. We assessed effect modification by including an interaction term between the interventions and pre-stratification factors in the Cox proportional hazards models. Effect modification was considered present when the interaction term had a *p* value of < 0.05. In addition, the interaction between IPS and PWI was modelled using Cox regression to determine whether the interaction was significant (which would indicate that the effect of the combination of IPS + PWI was different than the sum of IPS and PWI effects alone).

We also used log-rank tests and Cox regression analysis for the following secondary work-related outcomes: duration until any paid employment, duration until any paid employment or a trial placement or education, and duration until sustainable employment for at least three and for at least six months. Using nonparametric Mann–Whitney-U tests, the effect of the interventions on the total number of hours worked during follow-up was assessed. A mixed effect logistic regression analysis with the job coach as a random effect was performed to estimate the effect of each intervention on achieving any paid work during follow-up. GEE analyses with correction for baseline values were performed to assess the longitudinal effects of the interventions on the level of societal participation, mental health, physical health, ability to participate in social roles and activities, satisfaction with social roles and activities, and perceived work ability.

All analyses were applied according to the intention-to-treat (ITT) principle. In addition, per-protocol (PP) analyses were performed for the primary outcome. For the IPS intervention, we included clients in the per-protocol analysis if they were assigned to an IPS coach for at least one year (i.e. they were not referred to a different department or organisation within the first year after inclusion in the trial). For PWI, we determined that the intervention was performed according to protocol when at least the task analysis was performed with the employer and client.

For all analyses, we set the two-tailed significance level at *α* = 0.05. The analyses were performed in R (version 4.1.3) and SPSS (version 25 IBM, Armonk, NY, USA).

## Results

### Participant Flow and Dropout

**Fig. 1:**
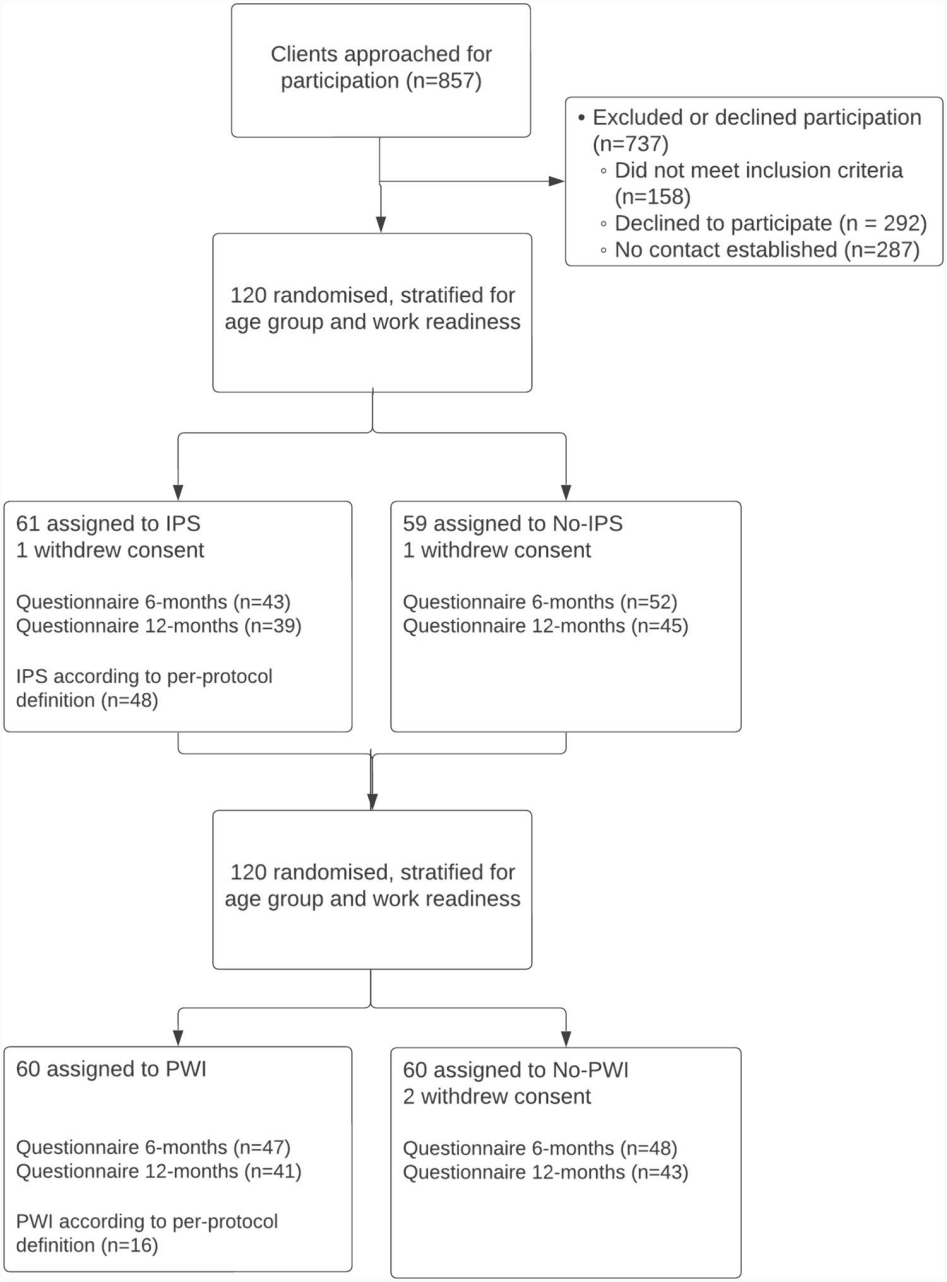
Flow diagram inclusion of participants

The flow of participants in the study is shown in Fig. 1. Of the 857 clients that were approached for participation, 287 (33%) could not be reached and 158 (18%) did not meet inclusion criteria. Of the remaining 412 clients that were eligible for participation 120 (29%) agreed to participate. Of these, 61 were assigned to IPS and 60 to PWI. Thirty-one clients received IPS and PWI. Because two participants withdrew their consent, data on the primary outcome and secondary outcomes that were collected from CBS was available for 118 clients at the 18-month follow-up. Ninety-five clients (81%) filled in the questionnaire at 6 month follow-up and 84 (71%) at 12-month follow-up.

### Participant Characteristics

Baseline characteristics of participants are shown in Table 1. Most clients were male (62%) and over 26 years old (63%). Over half of the clients were considered ‘ready to work’ (57%). Education level was low for the majority of clients (59%) and 7% were married or living with a partner. Most clients (79%) were very sure, sure or neutral regarding whether they would be able to work within six months after inclusion. There were no statistically significant differences between IPS and no-IPS and between PWI and no-PWI groups.

**Table 1:** Baseline characteristics of clients

	IPS		PWI	
	Yes (*N* = 60)	No (*N* = 58)	Yes (*N* = 60)	No (*N* = 58)
Age at inclusion (mean, SD)	34 (14)	37 (15)	35 (15)	36 (15)
Age group (N, %)				
Referred to adolescent team (16–26 years)	23 (38%)	22 (38%)	23 (38%)	22 (38%)
Referred to adult team (> 26 years)	37 (62%)	36 (62%)	37 (62%)	36 (62%)
Work readiness (N, %)				
Prevocational training required	25 (42%)	26 (45%)	27 (45%)	24 (41%)
Able to work directly	35 (58%)	32 (55%)	33 (55%)	34 (59%)
Gender (male) (N, %)	40 (67%)	34 (57%)	35 (58%)	39 (67%)
Duration of unemployment (N, %)				
Paid employment at baseline (< 12 h/week)	1 (2%)	4 (7%)	2 (3%)	3 (5%)
Max. 6 months	16 (27%)	13 (22%)	15 (25%)	14 (24%)
More than 6 months, less than a year	6 (10%)	5 (9%)	3 (5%)	8 (14%)
More than 1 year	24 (40%)	27 (47%)	28 (47%)	23 (40%)
Never employed	7 (12%)	6 (10%)	8 (13%)	5 (9%)
Missing	6 (10%)	3 (5%)	4 (7%)	5 (9%)
Marital status				
Single	(92%)	53 (91%)	(92%)	53 91%)
With partner	3 (5%)	4 (7%)	4 (7%)	3 (5%)
Missing	2 (3%)	1 (2%)	1 (2%)	2 (3%)
Living situation				
Homeless	1 (2%)	–	–	1(2%)
Living alone	21 (35%)	20 (34%)	20 (33%)	21 36%)
Living with family or friends	28 (47%)	33 (57%)	33 (55%)	28 48%)
Assisted living	4 (7%)	1 (2%)	1 (2%)	4 (7%)
Missing	6 (10%)	4 (7%)	6 (10%)	4 (7%)
Do you have children ≤ 5 years?				
Yes	2 (3%)	4 (7%)	2 (3%)	4 (7%)
No	57 (95%)	54 (93%)	58 (97%)	53 (91%)
Missing	1 (2%)	–	–	1 (2%)
Educational level^a^				
Lower	31 (52%)	39 (67%)	34 (57%)	36 62%)
Intermediate	14 (23%)	11 (19%)	12 (20%)	13 22%)
Higher	14 (23%)	8 (14%)	14 (23%)	8 (14%)
Missing	1 (2%)	–	–	1 (2%)
How sure are you that you will be able to work within 6 months?				
(very) sure or neither sure nor unsure	47 (78%)	46 (79%)	49 (82%)	44 (76%)
(very) unsure	11 (18%)	12 (21%)	10 (17%)	13 (22%)
Missing	2 (3%)	–	1 (2%)	1 (2%)

^a^Education level: lower = (special) primary education, first three years of general secondary education or pre-university education, prevocational education, and lower secondary vocational training; intermediate = upper secondary education, basic vocational training, vocational training and middle management and specialist education; higher = associate degree programs, higher education bachelor’s programs, master’s degree programs and doctoral degree programs

### Primary Outcome

**Fig. 2:**
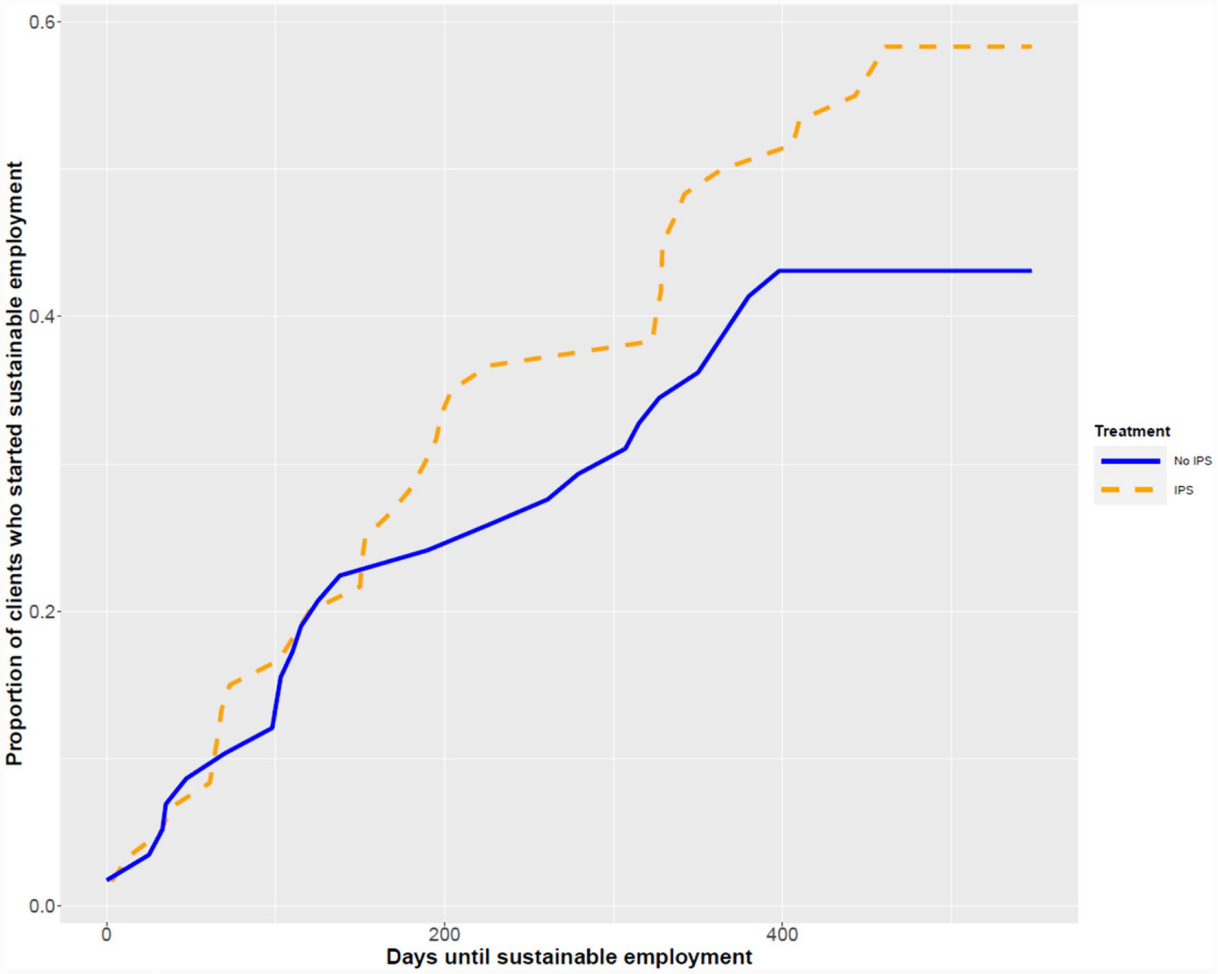
Survival curves for duration until sustainable paid employment for the IPS and no-IPS groups

**Fig. 3:**
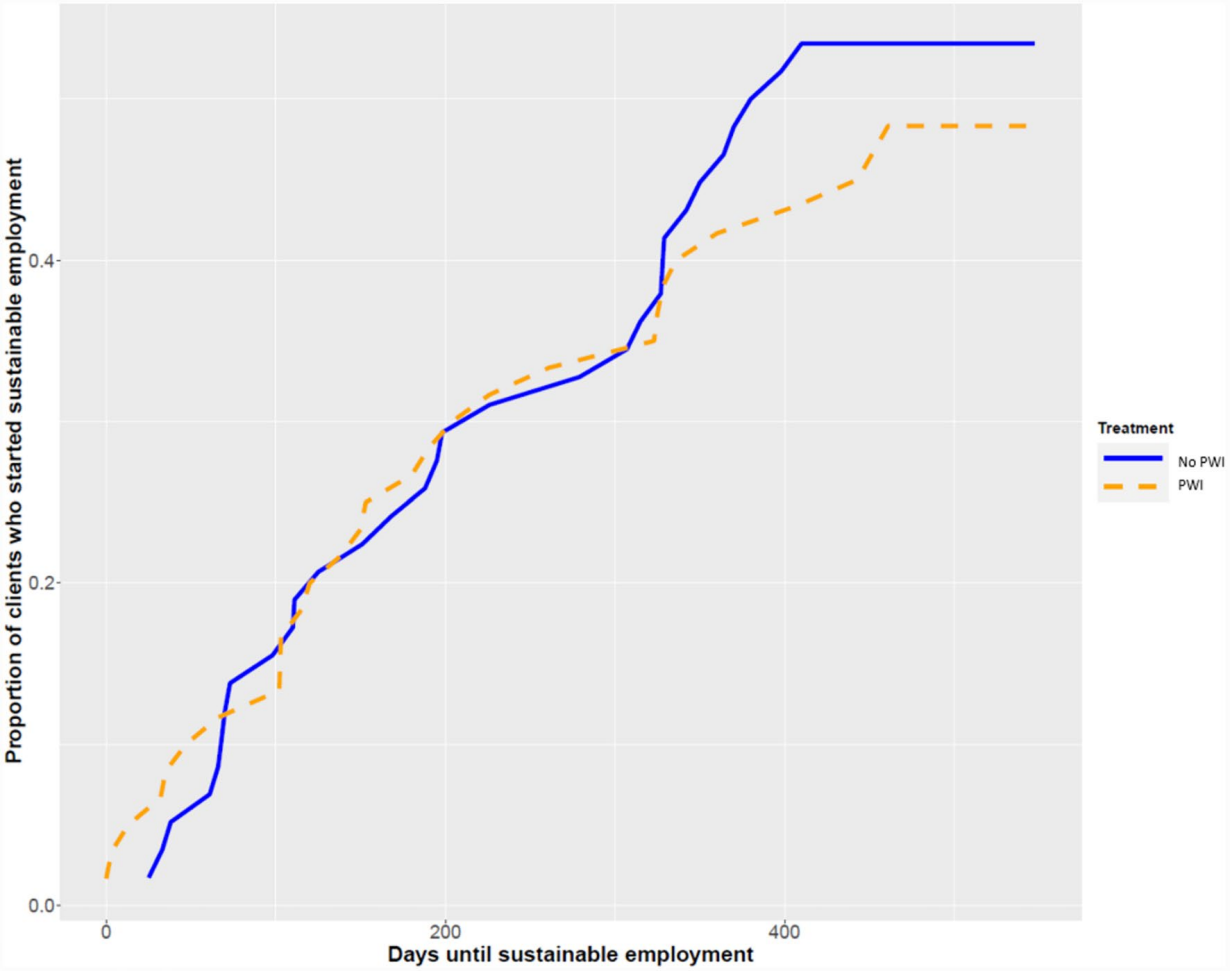
Survival curves for duration until starting sustainable paid employment for the PWI and no-PWI groups

Days until starting sustainable employment for the different study groups, including hazard ratios, are shown in Table 2. For the IPS group, RMST was 352 days and 394 days in the no-IPS group (HR = 1.47, 95% CI = 0.81–2.63). For the PWI group, RMST was 378 days and 394 days in the no-PWI group (HR = 0.89, 95% CI = 0.48–1.64). Figures 2 and 3 show the survival curves for both interventions. The p value of the interaction term between IPS and PWI was > 0.05, indicating there was no additional effect of IPS + PWI.

**Table 2:** Results of survival analyses for duration until sustainable paid employment (i.e. paid employment for at least 28 days and at least an average of 12 h/week)

	Kaplan–Meier (KM) analysis		Cox regression with robust standard errors	
	Restricted mean survival (unemployment) time	Logrank test *p*-value	HR	95% CI
No-IPS	394 (343;444)			
IPS	352 (302;402)	0.1	1.47	0.81;2.63
No-IPS	394 (343;444)			
IPS per-protocol	328 (274;381)	**0.03**	1.80	**1.03;3.14**
No-PWI	367 (318;417)			
PWI	378 (327;429)	0.6	0.89	0.48;1.64
No combined intervention	380 (338;421)			
Combined intervention	352 (283;420)	0.3	1.37	0.81;2.32

Statistically significant (α = 0.05) values are given in bold

#### Per-Protocol Analyses

For the IPS per-protocol analysis, 12 clients from the IPS group were excluded because they were not assigned to an IPS coach for at least 12 months. RMST was 328 days in the IPS per-protocol group, and 394 days in the no-IPS group (HR = 1.80, 95% CI = 1.03–3.14). We did not perform a per-protocol analysis for PWI, because PWI was only carried out according to protocol for 16 of the 60 participants (i.e. at least the task analysis was performed with the client and employer).

### Secondary Outcomes

No significant differences between the groups were found for most secondary employment-related outcomes. We found a positive effect of IPS compared to no-IPS for days until starting any work, a trial placement, or education; for this outcome RMST was 222 in the IPS group, and 335 in the no-IPS group (HR = 1.85, 95% CI = 1.01–3.42).

Mental and physical health, (satisfaction with) ability to participate in social roles and activities and societal participation remained more or less stable over time. For mental health we found a small but significant difference between PWI and no-PWI, with a lower score (-4.07) in the PWI group (95% CI = −7.93– −0.22). The results for all secondary outcomes can be found in Appendix 1 (supplementary file 1).

## Discussion

This study examined the effects of IPS and PWI on duration until starting sustainable paid employment for people with work disabilities. We found no significant differences in time until starting sustainable employment between IPS and no-IPS and between PWI and no-PWI. No interaction effect between IPS and PWI was found. Secondary outcomes showed that time until starting any paid employment, a trial placement, or education was significantly shorter for the IPS group than for the no-IPS group. Mental health scores in the PWI group were slightly lower than in the no-PWI group, indicating poorer mental health in the PWI group. No other significant differences were found for secondary outcomes.

In contrast to many previous studies, we did not find that IPS was more effective than no-IPS in increasing participation in paid work [14, 17, 39]. Even though all comparisons between IPS and no-IPS seem to indicate that IPS is superior to no-IPS, most differences were not statistically significant. The most likely explanation for our results is that we had insufficient power to prove differences. For our power calculation we used an expected hazard ratio of 2.0 based on previous research [17, 24]. In hindsight this may have been too high, because the proportion of clients who reached the primary outcome in the control groups in our study was larger than expected, due to differences in the design and setting of our trial. The observed HR was therefore lower than generally expected, which led to a decrease in statistical power.

There are multiple reasons why the proportion of clients in our control groups started work more often than expected. First, previous studies took place in mental healthcare settings (usually focussed on healthcare), while our study took place in a setting that is solely focussed on coaching people towards employment. This might explain the high proportion of clients who started work in the intervention and control groups. To illustrate, a previous Dutch study amongst people with SMI in the community mental health care setting found that 44% of clients in the IPS group and 25% of clients in the control group started work after 30 months [14]; in our study this was 70% of the clients in the IPS group and 55% of clients in the no-IPS group after 18 months.

Next, our target population is different from previous studies. Compared to people with SMI, it is possible that the symptoms and problems people in our study experienced were less hindering; and therefore, their a priori chance of starting work may have been larger. Previous research also showed that IPS is more effective for clients with SMI than for clients with CMD—a group perhaps more comparable to our target population [20].

A final reason for why the hazard ratio of 2.0 might have been too optimistic is that we used a factorial design. This likely led to less contrast between the IPS and no-IPS group than in previous research. Whereas in most IPS studies the control group received some form of prevocational training according to the ‘first train, then place’ principle, in our study most clients in the control group (approximately 75%) received coaching according to the ‘first place, then train’ principle. After all, the no-IPS group consisted of a combination of clients who received PWI and clients who received SAU. For clients who received PWI (± 50% of the no-IPS group) as well as for clients who received SAU and who were considered ready to work (± 25%), the focus was on finding paid employment from the start of the trajectory. Only clients who received SAU and who were considered not yet ready to work (± 25%) received coaching based on a ‘first train, then place’ principle.

Besides insufficient power, our findings might also be explained by an insufficient level of implementation, especially because our per-protocol analysis showed that IPS was superior to no-IPS. We also performed a process evaluation [40], which indeed showed that the level of implementations of IPS was sub-optimal and that the fidelity score was only 92 (“fair”) [41]. The main issue regarding fidelity was a lack of integration with healthcare services [40]. Because previous research has shown that a higher fidelity score corresponds with better employment outcomes, this might also in part explain why the observed differences between IPS and no-IPS were less than expected [42].

Finally, it must be noted that when we expanded the outcome to include the start of education and trial placements, IPS was superior to no-IPS. Because some clients in our study population preferred to start education instead of paid employment, it could be argued this is an important part of the outcome. The same goes for trial placements, which are often used in the setting that our study was performed in [30]. By using a trial placement, the employer and employee can get to know each other and both have a chance to more freely explore the suitability of the match. However, we do not have information regarding the extent to which education and trial placements lead to a paid job in the long-term; and thus, further research is necessary to explore whether these are suitable endpoints for IPS trajectories in this setting.

For PWI, we did not find significant differences on any of the work-related outcome measures, even though previous research showed that PWI can be an effective intervention in terms of increasing return to work [23, 24, 37]. However, our process evaluation showed that the implementation of PWI was low [40]. One reason for the low implementation is that the main part of PWI could only be carried out when the client had a workplace, which was not achieved for a large part of the clients in the PWI group. However if clients did find a job, the PWI conversation in the workplace between job coach, employee, and supervisor also only took place in less than 40% of cases [40]. Our results are thus in line with previous research on participatory workplace interventions, in which limited or no effects were found when the implementation of the intervention is low [25–27]. Therefore, further research is needed to address how the implementation can be improved and how this intervention can be effective in this specific context.

### Strengths and Limitations

This study has several strengths and limitations. One strength is that we performed an RCT, which ensured that even though the study took place over a prolonged period in which the labour market underwent many changes (including due to the COVID-19 pandemic and subsequent lockdowns), we could still make comparisons between groups and draw conclusions. Second, we used registry instead of self-reported data which resulted in complete and reliable data for the primary outcome for all participants, and thus eliminated bias due to missing data.

An important limitation is that we could not randomise job coaches, which may have led to bias. It is plausible that the job coaches we included were specifically motivated for the intervention they carried out and that therefore their adherence to protocol was higher than it would have been if they had not volunteered for a specific intervention. This may have led to an overestimation of the real effect size. Second, the participation rate of clients in our study was relatively low. Even though this low percentage is comparable to other studies involving similar populations [26, 43], there may have been some response bias which limited external validity, especially because clients who were not able to understand the informed consent form (either due to cognitive or language barriers) were excluded from participation. Therefore, the results might be less generalizable to the complete target population of people with work disabilities.

### Recommendations for Future Research

Further research with sufficient power and a higher level of implementation is necessary to assess the effectiveness of IPS and PWI in the municipal setting. It is important to explore how implementation could be improved, and a qualitative study among important stakeholders might give more insight into this matter. Because the goal of PWI is mainly to improve sustainability of employment, using a different primary outcome based on a different operationalization of sustainable employment (e.g. more than six months) and/or using a longer follow-up period might also show different results.

### Conclusion

Neither IPS nor PWI significantly decreased the duration until sustainable employment for unemployed people with work disabilities. Clients who received IPS started paid employment more often, but the differences we found were not statistically significant. When we expanded the outcome ‘time until starting any work’ by including education and trial placements, IPS did show significant beneficial effects. It is possible that IPS is indeed a promising intervention for this setting and population, but further research is necessary to prove effectiveness. We found no important differences for PWI, most probably due to a low level of implementation. Research on how to increase implementation of both IPS and PWI could therefore be valuable.

## Supplementary Information

Below is the link to the electronic supplementary material.

Supplementary file1 (PDF 689 KB)
