# Supplementary material for: Individual Placement and Support and Participatory Workplace Intervention on the Work Participation of People with Disabilities: A Randomised Controlled Trial
Source: J Occup Rehabil. 2024 Jul 2;35(3):520–32. doi: 10.1007/s10926-024-10219-6 (PMC12360975; doi:10.1007/s10926-024-10219-6)
Supplement: Supplementary file 1 — Supplementary file1 (PDF 689 KB) [file 10926_2024_10219_MOESM1_ESM.pdf]

Individual Placement and Support and Participatory Workplace Intervention on the Work Participation of People with Disabilities: A Randomised Controlled Trial

E. Oude Geerdink[1], M.A. Huysmans[1], H. van Kempen[2], J. van Weeghel[3], E. Motazedi[1] , J.R. Anema[1]

1- Department of Public and Occupational Health, Amsterdam Public Health Research Institute, Amsterdam UMC, Vrije Universiteit Amsterdam, Amsterdam, 1081 BT, Netherlands

2- Research and Statistics, City of Amsterdam, Amsterdam, The Netherlands

3- Tranzo, Tilburg School of Social and Behavioral Sciences, Tilburg University, Tilburg, The Netherlands

Appendix 1: Results for secondary outcomes

Table 3: Results of mixed effect logistic regression analyses for starting any paid employment

Outcome measure	Group	Outcome measure reached, N (%)	OR (95% CI)	p-value
Any paid employment	IPS vs. No-IPS<br>PWI vs. No-PWI	42 (70%) – 32 (55%)<br>34 (56%) – 40 (69%)	1,90 (0,89 – 4.04)<br>0,59 (0,28 – 1.25)	0.10<br>0.17

Table 4: Results of survival analyses for duration until starting any paid employment

	Kaplan-Meier (KM) analysis		HR from Cox regression with robust standard errors	
	Restricted mean survival (unemployment) time	Log-rank test p-value	HR	95% CI
No-IPS	382 (331;434)			
IPS	318 (266;369)	0.08	1.55	0.81;2.97
No-PWI	331 (280;383)			
PWI	367 (315;420)	0.2	0.73	0.37;1.44

Table 5: Results of survival analyses for duration until starting any paid employment, a trial placement, or regular education

	Kaplan-Meier (KM) analysis		HR from Cox regression with robust standard errors	
	Restricted mean survival (unemployment) time	Log-rank test p-value	HR	95% CI
No-IPS	335 (279;391)			
IPS	222 (173;270)	**0.006**	1.85	**1.01;3.42**
No-PWI	250 (198;301)			
PWI	304 (249;360)	0.1	0.73	0.36;1.46

Statistically significant (α = 0.05) values are highlighted in bold.

Table 6: Results of survival analyses for duration until starting sustainable paid employment for at least 3 months

	Kaplan-Meier (KM) analysis		HR from Cox regression with robust standard errors	
	Restricted mean survival (unemployment) time	Log-rank test p-value	HR	95% CI
No-IPS	434 (392;476)			
IPS	382 (333;430)	0.2	1.44	0.80;2.61
No-PWI	385 (339;432)			
PWI	428 (384;473)	0.1	0.63	0.35;1.12

Table 7: Results of survival analyses for duration until starting sustainable paid employment for at least 6 months

	Kaplan-Meier (KM) analysis		HR from Cox regression with robust standard errors	
	Restricted mean survival (unemployment) time	Log-rank test p-value	HR	95% CI
No-IPS	449 (406;491)			
IPS	395 (347;442)	0.1	1.63	0.92;2.87
No-PWI	411 (364;458)			
PWI	431 (386;475)	0.5	0.82	0.46;1.44

Table 8: Results of Mann-Whitney-U tests for total number of hours worked in paid employment

**Outcome measure**	**Group**	**Median (95% CI)**	**p-value**
Hours worked	IPS vs. No-IPS<br>PWI vs. No-PWI	798 (479, 1163) – 772 (416, 930)<br>696 (359, 1170) – 798 (537, 943)	0.27<br>0.90

Table 9: Results of GEE analyses, adjusted for the outcome values at baseline, for societal participation, perceived work ability, and health-related outcomes

**Outcome measure**	**Group**	**Mean difference per time unit (95% CI)**	**p-value**
Level of societal participation	IPS vs. No-IPS<br>PWI vs. No-PWI	-0.06 (-0.71, 0.58)<br>-0.55 (-1.18, 0,08)	0.85<br>0.09
Perceived work ability	IPS vs. No-IPS<br>PWI vs. No-PWI	-0.59 (-1.40, 0.22)<br>-0.53 (-1.33, 0.27)	0.16<br>0.20
**Outcome measure**	**Group**	**Mean difference (95% CI)**	**p-value**
Mental health	IPS vs. No-IPS<br>PWI vs. No-PWI	1.60 (-2.33, 5.54)<br>**-4.07 (-7.93, -0.22)**	0.43<br>**0.04**
Physical health	IPS vs. No-IPS<br>PWI vs. No-PWI	-1.13 (-3.89, 1.64)<br>-04e-04 (-2.71, 2.71)	0.43<br>1.00
Ability to participate in social roles and activities	IPS vs. No-IPS<br>PWI vs. No-PWI	-1.52 (-4.30, 1.26)<br>-1.27 (-4.08, 1.53)	0.29<br>0.38
Satisfaction with social roles and activities	IPS vs. No-IPS<br>PWI vs. No-PWI	1.33 (-1.78, 4.44)<br>-0.99 (-4.09, 2.11)	0.40<br>0.53

Statistically significant (α = 0.05) values are highlighted in bold.
